# Heat and Hypoxic Acclimation Increase Monocyte Heat Shock Protein 72 but Do Not Attenuate Inflammation following Hypoxic Exercise

**DOI:** 10.3389/fphys.2017.00811

**Published:** 2017-10-16

**Authors:** Ben J. Lee, Charles D. Thake

**Affiliations:** ^1^Occupational Performance Research Group, Department of Sport and Exercise Sciences, University of Chichester, Chichester, United Kingdom; ^2^Centre for Applied Biological and Exercise Sciences, Coventry University, Coventry, United Kingdom

**Keywords:** HSP72, I-FABP, Plasma cytokines, cross-tolerance, acclimation

## Abstract

Acclimation to heat or hypoxic stress activates the heat shock response and accumulation of cytoprotective heat shock proteins (HSPs). By inhibiting the NF-κB pathway HSP72 can preserve epithelial function and reduce systemic inflammation. The aim of this study was to determine the time course of mHSP72 accumulation during acclimation, and to assess intestinal barrier damage and systemic inflammation following hypoxic exercise. Three groups completed 10 × 60-min acclimation sessions (50% normoxic VO_2_peak) in control (*n* = 7; 18°C, 35% RH), hypoxic (*n* = 7; F_i_O_2_ = 0.14, 18°C, 35% RH), or hot (*n* = 7; 40°C, 25% RH) conditions. Tumor necrosis factor-α (TNF-α), interleukin 6 (IL-6), interleukin 10 (IL-10), and intestinal fatty acid binding protein (I-FABP) were determined at rest and following a cycling normoxic stress test (NST; ~2 weeks before acclimation), pre-acclimation hypoxic stress test (HST1; F_i_O_2_ = 0.14, both at 50% normoxic VO_2_peak; ~1 week before acclimation) and post-acclimation HST (48 h; HST2). Monocyte HSP72 (mHSP72) was determined before and after exercise on day 1, 3, 5, 6, and 10 of acclimation. Accumulation of basal mHSP72 was evident from day 5 (*p* < 0.05) of heat acclimation and increased further on day 6 (*p* < 0.01), and day 10 (*p* < 0.01). In contrast, basal mHSP72 was elevated on the final day of hypoxic acclimation (*p* < 0.05). Following the NST, plasma TNF-α (–0.11 ± 0.27 ng^.^mL^−1^), IL-6 (+0.62 ± 0.67 ng^.^mL^−1^) IL-10 (+1.09 ± 9.06 ng^.^mL^−1^) and I-FABP (+37.6 ± 112.8 pg^.^mL^−1^) exhibited minimal change. After HST1, IL-6 (+3.87 ± 2.56 ng^.^mL^−1^), IL-10 (+26.15 ± 26.06 ng^.^mL^−1^) and I-FABP (+183.7 ± 182.1 pg^.^mL^−1^) were elevated (*p* < 0.01), whereas TNF-α was unaltered (+0.08 ± 1.27; *p* > 0.05). A similar trend was observed after HST2, with IL-6 (+3.09 ± 1.30 ng^.^mL^−1^), IL-10 (+23.22 ± 21.67 ng^.^mL^−1^) and I-FABP (+145.9 ±123.2 pg^.^mL^−1^) increased from rest. Heat acclimation induces mHSP72 accumulation earlier and at a greater magnitude compared to matched work hypoxic acclimation, however neither acclimation regime attenuated the systemic cytokine response or intestinal damage following acute exercise in hypoxia.

## Introduction

Repeated exercise-heat exposures, leading to heat acclimation, can induce within lifetime phenotypic adjustments which enhance heat loss mechanisms and improve physiological responses to heat stress (Sawka et al., 2011; Horowitz, 2014). Heat acclimation improves tolerance to a diverse array of non-thermal stressors without a prior exposure to the new stressor-termed “cross tolerance” (Horowitz, 2016). Heat-acclimation mediated cross tolerance (HACT) to ischemia-reperfusion in the heart, brain hyperoxia, traumatic brain injury, and hypoxia have all been documented in animal models (Arieli et al., 2003; Horowitz, 2007; Shein et al., 2007) with increasing evidence for HACT in human models (Lee et al., 2014, 2015a, 2016; Gibson et al., 2015a,b; White et al., 2016).

Cross-tolerance effects are mediated by shared cellular adaptation, with the actions of the heat shock response (HSR) and upregulation of key heat shock protein family members being the most widely studied component of cross-tolerance in humans (Horowitz, 2016; Gibson et al., 2017). Heat acclimation is characterized as either having short term (STHA) and long term (LTHA) induction periods. STHA has been characterized as <5 daily heat exposures (Pandolf, 1979), and LTHA > 10 daily heat exposures (Garrett et al., 2011). Experimental evidence supports increases in basal heat shock protein 72 (HSP72) in response to exercise models of STHA; (Lee et al., 2015a), LTHA, (McClung et al., 2008; de Castro Magalhães et al., 2010; Gibson et al., 2015a,b; Lee et al., 2016) and both resting and exercising hypoxic exposures (Taylor et al., 2010, 2011, 2012; Lee et al., 2014). The enhancement of cytoprotective networks via progressive transcriptional activation and a buildup of HSP72 reserves occurs progressively throughout the acclimation period (Horowitz, 2016). Increased basal HSP72 is an integral adaptive mechanism that reduces the need for *de-novo* protein synthesis in response to later cellular stressors (Horowitz and Assadi, 2010; Horowitz, 2014, 2016). Heat acclimation has been shown to induce a greater daily physiological stress and increase in post-exercise and post-acclimation monocyte HSP72 compared to a matched absolute-intensity period of hypoxic acclimation (Lee et al., 2015a, 2016). This data suggests that heat *per se* is a more potent stimulator of the HSR and may be a more accessible method for enhancing both cellular and physiological tolerance. However, to date no study has examined the kinetics of the HSP72 response throughout the adaptive period of both heat and hypoxia.

Intracellular HSP72 (iHSP72) can induce anti-inflammatory effects by blocking nuclear factor-κB (NF-κB) activation, and the induction of iHSP72 via heat stimulation reduces the expression of inflammatory genes TNF-a, IL-1, IL-10, IL-12, and IL-18 (Ghosh et al., 1998). The HSR and blocking of NF-κB has been shown to enhance epithelial barrier resistance and reduces cytokine production through inhibition of NF-κB (Zuhl et al., 2014, 2015). Cellular injury to the intestinal tract has been observed followed a multitude of physiological stressors (heat stress, hypoperfusion/ischemia, oxidative stress, mechanical damage), which contribute to intestinal barrier disturbances (Pires et al., 2016; March et al., 2017). During exercise-heat stress a loss of epithelial integrity, increased GI permeability, and bacterial translocation induce a strong inflammatory response, which is characterized by activation, production, and release of cytokines, which are linked to exertional heat stroke (Bouchama and Knochel, 2002; Leon and Helwig, 2010). Acute hypoxia (<2 h at 4,800 m) has been shown to reduce splanchnic perfusion via a reduction in blood flow to the superior mesenteric artery (Loshbaugh et al., 2006), however this finding is not consistent across studies (Mekjavic et al., 2016), with perfusion increasing during prolonged (3 day) exposure (Kalson et al., 2010). However, an acute reduction in oxygen availability may lead to intestinal cell damage and a loss of epithelial integrity (Ohri et al., 1994; Derikx et al., 2008). A hypoxic stimulus combined with intestinal ischemia activates NF-κB in intestinal epithelial cells, increasing the production of the pro-inflammatory cytokine tumor necrosis factor alpha (TNF-α) (Eltzschig and Carmeliet, 2011). It is therefore plausible that heat or hypoxic-acclimation mediated activation of the HSR and inhibition of the NF-κB pathway may attenuate intestinal damage and reduce the inflammatory response following a later bout of exercise in acute hypoxia.

The aim of this study was to examine the rate of basal HSP72 accumulation throughout a 10-day period of exercising heat or hypoxic acclimation. We hypothesized that both modes of exercise-acclimation would elevate resting monocyte HSP72, and that this would be reflected in a shift toward an anti-inflammatory status as determined via eHSP72–mHSP72 ratio (*R;* Krause et al., 2015), and change in systemic cytokine balance (TNF-α, IL-6, IL-10). A secondary aim of the present study was to determine whether a period of heat or hypoxic acclimation would attenuate intestinal damage and reduce systemic inflammation following acute hypoxic exercise.

## Methods

This study is part of a recently published larger study of heat acclimation and hypoxic performance (Lee et al., 2016). However, this study focuses on the inflammatory response and intestinal cellular damage following a hypoxic exercise bout completed after acclimation to either heat or hypoxia.

### Participants

Participants (*n* = 21 males; age 22 ± 5 years; stature 1.76 ± 0.07 m; mass 71.8 ± 7.9 kg; VO_2_ peak 51 ± 7 mL^.^kg^−1.^min^−1^) provided written informed consent to take part in the study, which was approved by the Coventry University Ethics review panel. Participants were asked not to undertake any other exercise training in the 72 h leading up to a testing bout and throughout the intervention period and were blinded to the hypotheses of the study. All data collection was conducted in accordance with the standards set out in the Declaration of Helsinki of 1996.

### Study design

**Figure 1:**
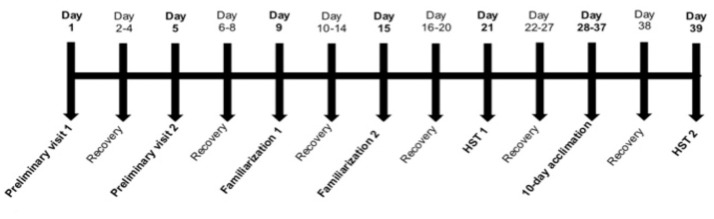
Schematic of the experimental design. Participants attended the laboratory a total of 16 times over ~39 days. Preliminary assessments of normoxic and hypoxic $\overset{\cdot }{V}$O_2_peak were performed in a counterbalanced order separated by at least 3 days. After at least 3 days' recovery (range 3–7 days) participants completed a full familiarization visit under normoxic conditions, followed 6 days (range 6–11) later by a second familiarization, from which the normoxic stress test (NST) data was obtained. At least 6 days after the NST (range 6–9 days), participants completed the pre-acclimation hypoxic stress test (HST1). Following a “wash out” period of 7 days (range 7–11 days), participants commenced 10 consecutive days of exercise acclimation. A second, post-acclimation HST (HST2) was performed 48 h after the final acclimation session in all participants.

As depicted in Figure 1, participants completed two preliminary assessments of normoxic and hypoxic $\overset{\cdot }{V}$O_2_peak, followed by a familiarization visit, a normoxic stress test (NST) and a hypoxic stress test (HST1). At least 7 days after HST1, participants completed a 10-day heat (*n* = 7), hypoxic (*n* = 7), or control (*n* = 7) acclimation period. A final HST (HST2) was conducted 48 h after the final acclimation session. On each familiarization, NST and HST session, as well as throughout the acclimation period, participants reported to the laboratory after an overnight fast to consume a standardized breakfast 2 h prior to the exercise bout. The energy content of the breakfast was 386kcal, made up of 15.6 g protein, 44.4 g carbohydrate and 16.4 g fat. Participants drank 400 ml of water with the breakfast. Each familiarization session, NST, HST, and acclimation session was preceded by 15 min of seated normoxic rest (after instrumentation) to collect baseline data and an additional 15 min of seated rest within the defined environment.

The familiarization, NST and HST sessions each consisted of 40 min of cycle exercise at 50% normoxic $\overset{\cdot }{V}$O_2_peak (CON = 137 ± 28 Watts; HYP = 138 ± 18 Watts; HOT = 136 ± 16 Watts), and a 5 min recovery period during which a venous blood sample was obtained for the assessment of mHSP72, TNF-α, IL-6, IL-10, and I-FABP. The 10-day acclimation protocol consisted of once daily exposures of cycle ergometer exercise (60 min) within the defined environment, either CON (18°C, 35% RH), HOT (40°C, 25% RH) or HYP (18°C, 35% RH, F_I_O_2_ = 0.14%) and performed at the same absolute power outputs described above (Castle et al., 2011). The control and hypoxic groups were blinded to their treatment groups by each breathing via a “hypoxic” reservoir filled with either hypoxic gas, or room air. Blinding to condition was not achievable for the heat group.

### Preliminary visits

Height was measured in the Frankfurt plane using a Harpenden stadiometer (Harpenden Instruments, Burgess Hill, UK), nude body mass determined on an electronic scale (Seca Body, Cranlea and Company, Birmingham, UK) and sum of skinfolds determined from 4 sites using a skinfold caliper (Harpenden Instruments, Burgess Hill, UK) as described by Durnin and Womersley (1974).

Peak $\overset{\cdot }{V}$O_2_ was determined in both normoxic and hypoxic conditions on separate days (preliminary visits 1 and 2) using an incremental exercise test to volitional exhaustion on a calibrated SRM cycle ergometer (Schoberer Rad Meßtechnik, Welldorf, Germany). Hypoxia was generated via a Hypoxicator unit (Hypoxico HYP123 Hypoxicator, New York, USA), that was used to fill a reservoir of three 1,000 L Douglas bags in series. Participants inspired via a mouthpiece attached to a two-way non-rebreathable valve (Harvard Ltd, Eldenbridge, UK) connected to the gas reservoir with clear ethylene vinyl tubing. Inspired oxygen content was monitored throughout each trial via a Servomex 1,400 gas analyzer (Servomex, Crowborough, UK) and sample line introduced through the Douglas bag sample port.

Resting blood lactate (BLa; Biosen C-Line analyser, EKF Diagnostics, Sailauf, Germany) was determined from a finger capillary whole blood sample following a 10-min seated rest period. The test began at a workload of 70 W for 4 min and was then increased by 35 W every 4 min until a blood lactate value of >4 mmol·L^−1^ was reached. Thereafter, workload increased 35 W every 2 min until volitional exhaustion. A cadence of 70 rev·min^−1^ was maintained throughout. Expired gases were collected using 200 L Douglas bags (Cranlea & Co, Birmingham, UK) during the final minute of each stage. Heart rate (Polar FT1, Polar Electro OY, Kempele, Finland) and perceived exertion (Borg, 1982) were recorded at the end of each gas collection. Respiratory gas analysis was completed as previously described (Lee et al., 2014, 2015a).

### Measurements

Prior to each testing session participants provided a urine sample for the assessment of urine specific gravity (USG; Atago Refractomer, Jencons Pls, Leighton Buzzard, UK) and urine osmolality (U_OSMO_; Advanced 3,300 Micro-Osmometer, Advanced Inc, Massachusetts, USA), determined their nude body mass (Seca, Bodycare, UK) and inserted a rectal thermistor (Grant Instruments, UK) to a depth of 10 cm. Heart rate (HR) was monitored throughout each trial via telemetry (Suunto, T6c, Finland). Blood lactate (Biosen C-Line analyser, EKF Diagnostics, Sailauf, Germany) was determined from a finger capillary whole blood sample at the end of the resting period and at the end of exercise for both HST and acclimation sessions.

During all hypoxic sessions, arterial oxygen hemoglobin saturation (S_P_O_2_) was measured throughout via a pulse oximeter (WristOx, Nonin Medical Inc, Minnesota, USA). Ratings of perceived exertion (RPE; Borg, 1982) and thermal sensation (TS; Young et al., 1987) were collected at 10 min intervals during the 40 min exercise tolerance phase of the test session with the mean exercise value reported.

### Blood sampling and analysis

Venous blood samples (~7 mL) were collected from an antecubital vein into an EDTA treated vacutainer (Vacuette, Greiner Bio-One, Stonehouse, UK) following the 15 min seated stabilization period before the familiarization session and each HST. Post-exercise samples were collected immediately after the exercise phase of the 60 min HST exposure was completed. Hemoglobin and haematocrit were determined in triplicate via a B-Hemoglobin Photometer (Hemocue Ltd, Angleholm, Sweden) and centrifuged capillary tubes (Hawksley Micro Haematocrit Centrifuge, Hawksley and Son, Lancing, UK), measured using a haematocrit reader. Plasma volume changes were then calculated according to the equations of Dill and Costill (1974). All later analysis for TNF-α, IL-6, IL-10, and I-FABP were corrected for any changes in plasma volume from pre to post-exercise. The remaining blood sample was centrifuged for 10 min at 3,000 RPM and plasma aliquoted for storage at −80°C prior to analysis.

Circulating TNF-a, IL-6, and IL-10 were determined in duplicate using commercially available high sensitivity sandwich ELISA kits (R&D systems, Minneapolis, USA) which were sensitive to 0.19 pg^.^mL^−1^, 0.18 pg^.^mL^−1^ 0.11 pg^.^mL^−1^ and respectively. The inter-assay variability for TNF-a, IL-6 and IL-10 was 3.1, 1.5, and 1.8%, respectively. I-FABP concentration was determined in duplicate via an ELISA kit (Hycult, Biotechnology, Uden, The Netherlands). Circulating eHSP70 was assessed using the commercially available Amp'd® HSP70 high sensitivity ELISA kit (ENZ-KIT-101-001; Lee et al., 2015b) according to the manufacturer's instructions (Enzo Lifesciences, Lausen, Switzerland). The ENZ-KIT is sensitive to 0.007 ng^.^mL^−1^ with a working range of 0.039–5.00 ng^.^mL^−1^.

### mHSP72 determination

An IgG1 isotype and concentration-matched FITC-conjugated negative control were used in order to assess non-specific binding. Briefly, cells obtained after red cell lysis were fixed and permeabilised (AbD Serotec, Kidlington, UK) and a negative control (FITC, AbD Serotec, Kidlington, UK) or anti-HSP72 antibody (SPA-810, Enzo lifesciences, Exeter, UK) was added to a final concentration of 100 μg·ml^−1^, this was used to label 1 × 10^6^ cells according to the manufacturer's instructions and then incubated for 30 min in the dark. Samples were then analyzed on a BD FACSCalibur (BD Biosciences, Oxford, UK) by flow cytometry with monocytes gated for forward/side scatter properties and further discriminated by CD14 expression (Selkirk et al., 2009). Mean florescence intensity (MFI) was then calculated using CellQuest Pro software (BD Biosciences, Oxford, UK) with a total of 15,000 cells counted.

### eHSP72 to iHSP72 ratio

The ratio of eHSP72 to iHSP72 was determined by dividing eHSP72 by iHSP72 (Krause et al., 2015). An increase in the ratio score was classed as a transition to a pro-inflammatory state, and a decrease in ratio score classed as a transition to an anti-inflammatory state.

### Statistical analysis

The primary outcome measure of this study were an assessment of the monocyte HSP72 response throughout the 10-day acclimation period. Secondary outcomes were the eHSP72, IL-6, IL-10, TNF-α, I-FABP responses before and after exercise on day 1 and day 10 of the acclimation period. Three families of hypotheses were tested according to the method of Benjamini and Hochberg (1995); (1) the absolute and percent changes in resting and post-exercise mHSP72 responses throughout acclimation; (2) the absolute and percent changes in eHSP72, TNF-α, IL-6, IL-10, and I-FABP before and after exercise; and (3) eHSP72/mHSP72 ratio before and after the acclimation period. The resting, mean exercise, and peak exercise physiological data have been reported in detail elsewhere (Lee et al., 2016) and are briefly presented over the course of acclimation for descriptive purposes. Mixed linear modeling with fixed effect for group (3), day (5) and time (2) was used to analyze mHSP72 data throughout the acclimation period. The same approach was used to analyze eHSP72, IL-6 and IL-10 before and after exercise on days 1 and day 10, with fixed effects for group (3), time (4) and day (2). In each instance main effects were explored using Tukey's HSD test and *post-hoc* results shown as *p* < 0.05 or *p* < 0.01. Precise *p*-values for main effects are reported alongside Cohen's D effect sizes (95% confidence intervals) to indicate the magnitude of any observed effects (Colquhoun, 2014). Effect sizes of 0.2, 0.5, and 0.8 are considered small, medium and large, respectively.

## Results

For complete performance results of the experimental and control groups, see our previously published study (Lee et al., 2016). All participants completed the full 60-min exercise bout during the acclimation period. Table 1 illustrates mean-exercise and peak-exercise data for heart rate, rectal temperature, physiological strain index, SpO_2_, change in body mass and change in plasma volume for days 1, 3, 5, 6, and 10 of the control, heat, and hypoxic acclimation period.

**Table 1:** Mean and peak exercise physiological responses throughout the 10-day acclimation period in the CON (*n* = 7), HYP (*n* = 7), and HOT (*n* = 7) experimental groups.

	**Mean exercise SpO_2_ (%)**	**Mean HR (bts^.^min^−1^)**	**Peak HR (bts^.^min^−1^)**	**Mean exercise T_rectal_ (°C)**	**Peak T_rectal_ (°C)**	**Mean PSI (AU)**	**Peak PSI (AU)**	**Change in Body mass (kg)**	**Change in PV (%)**
**CON**									
Day 1	98 ± 1	133 ± 21	137 ± 10	37.73 ± 0.27	37.86 ± 0.24	3.5 ± 1.3	4.0 ± 1.0	0.6 ± 0.2	−
Day 3	98 ± 1	133 ± 11	139 ± 14	37.74 ± 0.28	37.99 ± 0.24	3.8 ± 0.9	4.7 ± 0.6	0.6 ± 0.2	0.2 ± 4.7
Day 5	97 ± 1	129 ± 12	131 ± 17	37.61 ± 0.22	37.85 ± 0.09	4.3 ± 1.2	3.9 ± 1.1	0.9 ± 0.3	−1.0 ± 4.4
Day 6	98 ± 1	130 ± 10	136 ± 11	37.65 ± 0.22	37.89 ± 0.22	3.6 ± 0.9	4.5 ± 1.0	0.8 ± 0.3	3.3 ± 4.6
Day 10	98 ± 1	134 ± 12	137 ± 17	37.70 ± 0.28	37.86 ± 0.21	3.7 ± 0.9	4.2 ± 1.3	0.7 ± 0.3	2.4 ± 4.6
**HYP**									
Day 1	81 ± 2^b^^,^^f^	149 ± 16^a^	159 ± 15^b^	37.82 ± 0.51	38.16 ± 0.46	4.7 ± 1.5	5.9 ± 1.5^b^	0.8 ± 0.3	−
Day 3	81 ± 2^b^^,^^f^	151 ± 11^a^	157 ± 10^b^	37.89 ± 0.37	38.14 ± 0.29	5.2 ± 1.2^a^	6.0 ± 1.0^a^	0.7 ± 0.4	−2.9 ± 3.8
Day 5	81 ± 2^b^^,^^f^	150 ± 13^a^	157 ± 12^b^	37.84 ± 0.40	38.16 ± 0.38	5.2 ± 1.2^a^	6.2 ± 1.0^b^	0.7 ± 0.2	−3.1 ± 4.6^a^
Day 6	82 ± 2^b^^,^^f^	148 ± 11^a^	154 ± 8^b^	37.90 ± 0.39	38.20 ± 0.25	4.4 ± 1.3	5.3 ± 1.1	0.7 ± 0.4	−3.6 ± 3.6^a^
Day 10	82 ± 3^b^^,^^f^	136 ± 11^*^	142 ± 7^*^	37.66 ± 0.33	37.92 ± 0.22	4.1 ± 0.9	4.9 ± 0.5^*^	0.9 ± 0.3	−5.6 ± 3.7^*^
**HOT**									
Day 1	97 ± 1	151 ± 16^a^	164 ± 11^b^	37.91 ± 0.44	38.68 ± 0.26^c^^,^^e^	5.0 ± 1.7^a^	7.3 ± 1.2^c^^,^^e^	1.0 ± 0.6	−
Day 3	98 ± 2	145 ± 16^a^	157 ± 16^b^	37.81 ± 0.49	38.22 ± 0.38^#^	4.7 ± 1.4	6.2 ± 1.1^#^^,^^b^	1.2 ± 0.5	2.9 ± 1.7
Day 5	97 ± 1	147 ± 12^a^	156 ± 12^b^	37.86 ± 0.44	38.26 ± 0.37^#^^,^^b^	4.9 ± 1.3^a^	6.2 ± 1.1^*^^,^^b^	1.1 ± 0.3	4.3 ± 2.3
Day 6	98 ± 2	143 ± 13^*^^a^	150 ± 8^*^	37.77 ± 0.41	38.18 ± 0.29^#^	4.9 ± 1.3	6.2 ± 1.1^#^	1.5 ± 0.3^*^^b^^,^^d^	7.5 ± 3.6^*^
Day 10	98 ± 2	137 ± 14^*^	144 ± 9^#^	37.71 ± 0.33	38.06 ± 0.22^¥^	4.2 ± 1.0^*^	5.4 ± 0.5^¥^	1.9 ± 0.3^*^^,^^b^^,^^d^	8.3 ± 3.5^*^

Data are mean ± SD. Within group differences to day 1 of acclimation are denoted by

* *p < 0.05*,

# *p < 0.01*,

¥ p < 0.0001. Within day differences to the control condition are denoted by

a *p < 0.05*,

b *p < 0.01*,

c p < 0.0001. Within day differences to hypoxia are denoted by

d *p < 0.05*,

e p < 0.001. Within day differences to hot are denoted by

f *p < 0.0001*.

### Intracellular and extracellular responses to acclimation

**Figure 2:**
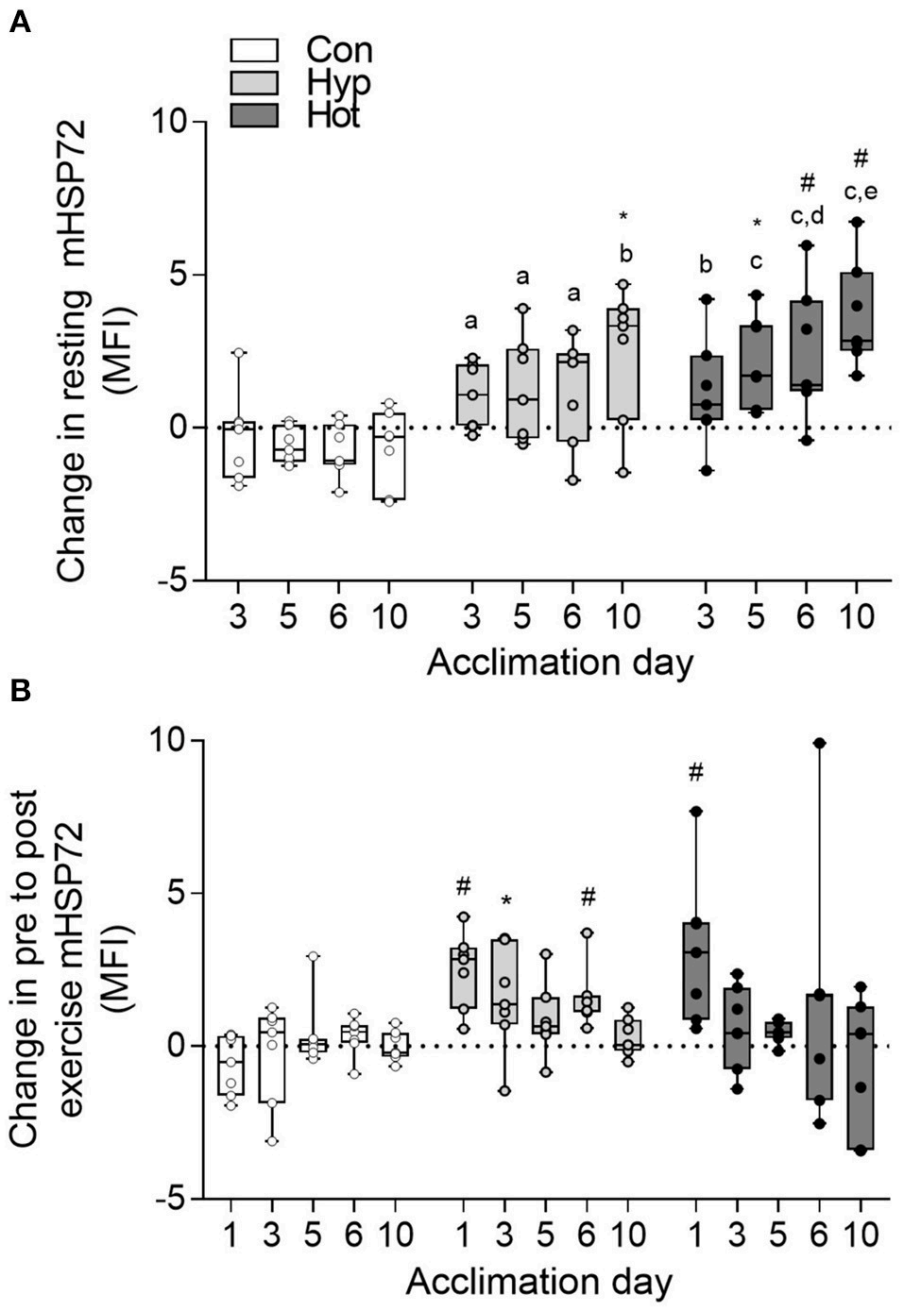
Delta change in resting monocyte HSP72 MFI relative to the resting value obtained on day 1 of acclimation **(A)**; and delta change in pre to post-exercise mHSP72 MFI relative to daily resting mHSP72 MFI **(B)**. Box plots show all individual data points (dots), the 25 and 75th interquartile ranges (boxes), and the median (mid-line). Whiskers illustrate the highest and lowest value. Letters illustrate changes from the resting mHSP72 value obtained on day 1 of acclimation between experimental groups. a, b, c illustrates *p* < 0.01, < 0.001, and < 0.0001 compared to control respectively; d and e illustrate *p* < 0.01 and < 0.001 compared to hypoxia respectively. Within group changes in mHSP72 relative to the resting mHSP72 value obtained on day one of acclimation **(A)** or pre to post-exercise **(B)** are illustrated by ^*^p < 0.05 and #*p* < 0.01).

No changes in mHSP72 or eHSP72 were observed in the control group at any phase of the acclimation period. Both hypoxic and heat acclimation increased resting concentrations of mHSP72 (time × trial interaction, *f* = 2.55, *p* = 0.003; Figure 2A). Specifically, basal mHSP72 MFI was increased in relation to day 1 on days 5 (*p* < 0.05; *d* = 2.1, 95% *CI* = 0.7–3.2), 6 (*p* < 0.05; *d* = 2.2, 95% *CI* = 0.8–3.4) and 10 (*p* < 0.01, *d* = 3.1, 95% *CI* = 1.4–4.4) in the heat acclimation group, and on day 10 in hypoxic group (*p* < 0.05; *d* = 1.3, 95% *CI* = 0.04–2.3). A post-exercise increase in mHSP72 was observed on day 1 of heat acclimation (*p* < 0.01), and on days 1 (*p* < 0.05), day 3 (*p* = 0.01), and day 6 (*p* < 0.01) of hypoxic acclimation (Figure 2B). A negative correlation between the recorded resting mHSP72 and fold change following exercise in both the heat (*r* = −0.87, *p* = 0.009) and hypoxic (*r* = −0.52, *p* = 0.22) groups was observed. A similar relationship was observed post-exercise on day of the hypoxic acclimation period (*r* = −0.55, *p* = 0.20), whereas a much weaker relationship between resting mHSP72 and the fold change post-exercise was observed after the heat acclimation period (*r* = −0.05, *p* = 0.92).

**Figure 3:**
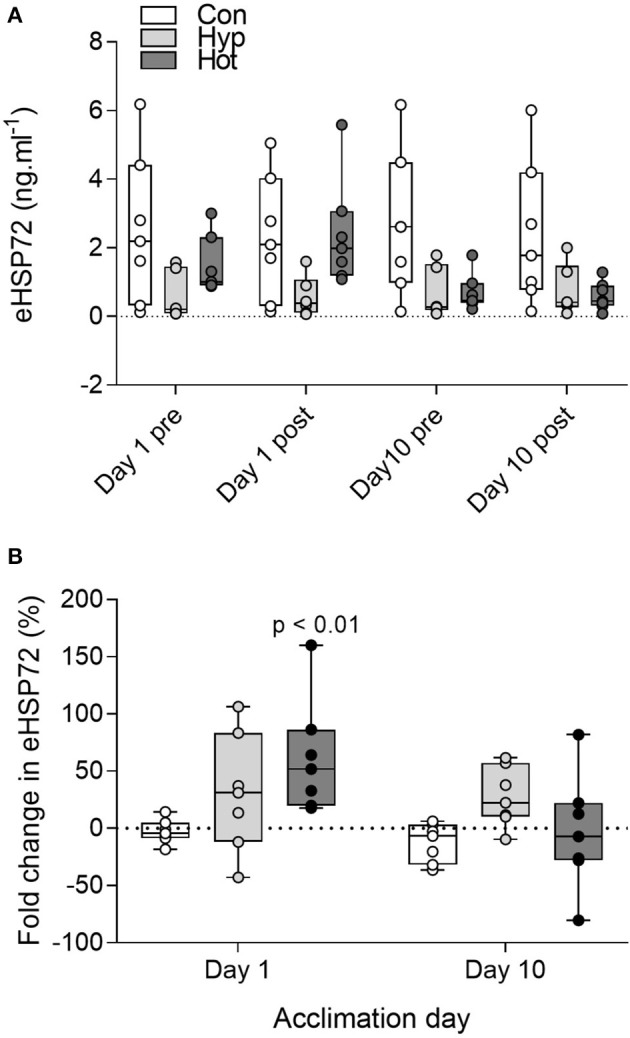
Extracellular HSP72 before and after exercise on day 1 and day 10 of the acclimation intervention for all individual participants **(A)**; and percent change in eHSP72 from pre to post-exercise on day 1 and day 10 of acclimation **(B)**. Box plots show all individual data points (dots), the 25 and 75th interquartile ranges (boxes), and the median (mid-line). Whiskers illustrate the highest and lowest value. For display purposes one participant with high eHSP72 values is not included in panel A (day 1 pre, 17.8 ng^.^mL^−1^, day 1 post, 24.4 ng^.^mL^−1^; Day 10 pre, 15.6 ng^.^mL^−1^, Day 10 post, 19.1 ng^.^mL^−1^). *P*-values indicate differences relative to pre-trial resting values within each experimental group **(B)**.

No main effects were observed for absolute eHSP72 concentrations (group *f* = 1.52, *p* = 0.23; time *f* = 0.14, *p* = 0.71; acclimation day *f* = 0.38, *p* = 0.54; Figure 3A). When reported as post-exercise percent change (Figure 3B), heat induced a 62 ± 50% (range 18–160%; *p* < 0.01, *d* = 0.7, 95% *CI* = −0.4–1.8) increase in eHSP72 following exercise on day 1 of acclimation, whereas hypoxia (31 ± 52%, range −43–106%, *d* = 0.1, 95% *CI* = −0.9–1.2) and control exercise (–2 ± 10.4%, range −18–14%, *d* = −0.1, 95% *CI* = −1.2–1.0) had little effect on eHSP72 concentrations. Following heat-acclimation the post-exercise fold change in eHSP72 was attenuated (−3.6 ± 50%, range −80–82%). The ratio of eHSP72 to mHSP72 was no different on day 10 (*R* = 0.47) of acclimation compared to day 1 (*R* = 0.54) in the control group and hypoxic group (day 1, *R* = 0.93; day 10, *R* = 0.48), but was reduced in the heat group (day 1, *R* = 0.28; day 10, *R* = 0.07; *p* = 0.007).

### Cytokine and I-FABP responses to acclimation–day 1

Cytokine data for before and after exercise on day 1 and day 10 of acclimation are shown in Table 2. Plasma IL-6 concentration increased with exercise on day 1 of acclimation in the HOT (+ 5.01 [2.78–7.24]′ ng^.^mL^−1^; *p* = 0.015) and HYP (+ 3.44 [2.43–4.45] ng^.^mL^−1^, *p* = 0.003) groups, with minimal changes observed after CON (+ 0.84 [0.09–1.60] ng^.^mL^−1^, *p* = 0.216). Plasma IL-10 also increased with exercise in the HOT (+ 67.14 [35.46–98.81] ng^.^mL^−1^; *p* < 0.001) and HYP (+ 24.17 [9.63–38.71] ng^.^mL^−1^; *p* < 0.001) groups, and was minimally affected after exercise in CON (+ 3.21 [−0.54–6.95] ng^.^mL^−1^, *p* = 0.145). TNF-α was undetectable at rest in 4/21 participants (1 CON, 1 HYP and 2 HOT) and unaffected by exercise in all groups at all phases of the study (Table 2). Plasma I-FABP concentrations were unaffected following exercise in CON (+ 28 [–61–118] pg^.^mL^−1^, *p* = 0.56), and was increased from resting values after HYP (+201 [128–273] pg^.^mL^−1^, *p* = 0.0013) and HOT (+282 [157–406], *p* = 0.004).

**Table 2:** Mean ± SD differences before and after exercise for systemic cytokines and intestinal fatty acid binding protein on day 1 and day 10 of the acclimation period.

	**Day 1**		**Day 10**	
**Variable**	**Before exercise**	**After exercise**	**Before exercise**	**After exercise**
**IL-6 (ng**^.^**mL**^−1^**)**				
CON	1.18 ± 0.65	2.02 ± 1.40	1.27 ± 0.60	2.21 ± 1.67
HYP	1.06 ± 0.84	4.49 ± 1.54^#^	1.10 ± 0.73	4.62 ± 1.74^*^
HOT	1.20 ± 0.81	6.22 ± 3.23^*^	1.26 ± 1.35	4.00 ± 2.72^*^^,^^a^
**IL-10 (ng**^.^**mL**^−1^**)**				
CON	3.61 ± 2.32	6.82 ± 5.39	3.21 ± 2.44	6.77 ± 4.53
HYP	12.39 ± 19.52	36.87 ± 26.35^*^	11.03 ± 14.60	39.97 ± 37.30^#^
HOT	18.56 ± 28.51	85.70 ± 59.35^#^	21.43 ± 36.66	53.71 ± 40.84^#^
**TNF-** α **(ng**^.^**mL**^−1^**)**				
CON	0.79 ± 0.62	1.26 ± 0.61	0.75 ± 0.39	0.40 ± 0.18
HYP	3.73 ± 7.30	4.32 ± 7.04	1.32 ± 1.02	0.97 ± 0.65
HOT	5.33 ± 6.16	4.88 ± 6.17	4.14 ± 7.71	4.16 ± 3.35
**I-FABP (pg**^.^**mL**^−1^**)**				
CON	352.3 ± 249.0	380.5 ± 240.0	331.6 ± 242.8	405.3 ± 202.1
HYP	449.6 ± 234.2	643.9 ± 232.3^#^	440.3 ± 179.5	651.7 ± 178.9^#^
HOT	370.1 ± 392.4	652.7 ± 100.4^#^	345.2 ± 350.8	554.7 ± 143.9^#^

Within group differences from pre to post-exercise are denoted by

* *p < 0.05*,

# < 0.01. Within group differences between trials are denoted by

a *p < 0.05. IL-6; Interleukin-6, IL-10; Interleukin-10, TNF-; Tumor necrosis factor-alpha, I-FABP; Intestinal fatty acid binding protein*.

### Cytokine and I-FABP responses to acclimation–day 10

The plasma IL-6 response to exercise remained consistent with that observed on day 1 of acclimation in the CON and HYP groups, with post-exercise IL-6 concentrations increasing by 0.95 [−0.29–2.18] and 3.52 [2.49–4.55] ng^.^mL^−1^ respectively. Moderate evidence for a decrease in post-exercise IL-6 concentration was observed for the HOT group (+ 2.74 [1.03–4.44] ng^.^mL^−1^, *p* = 0.036, vs. day 1). A similar pre to post-exercise change for IL-10 was observed in the CON (+ 3.56 [0.75–6.39] ng^.^mL^−1^) and HYP groups (+28.94 [6.75–51.14] ng^.^mL^−1^). In contrast to day 1, weak evidence for a reduction in post-exercise IL-10 concentrations were observed in the HOT group (+32.28 [7.61–56.95] *p* = 0.058, vs. day 1). Post-exercise I-FABP concentrations were unaffected by 10 days of CON (+73 [−54–201] pg^.^mL^−1^), HYP (+211 [155–267 pg^.^mL^−1^) or HOT (+209 [51–368], *p* = 0.16) exercise acclimation.

### Cytokine and I-FABP responses to hypoxic exercise

**Figure 4:**
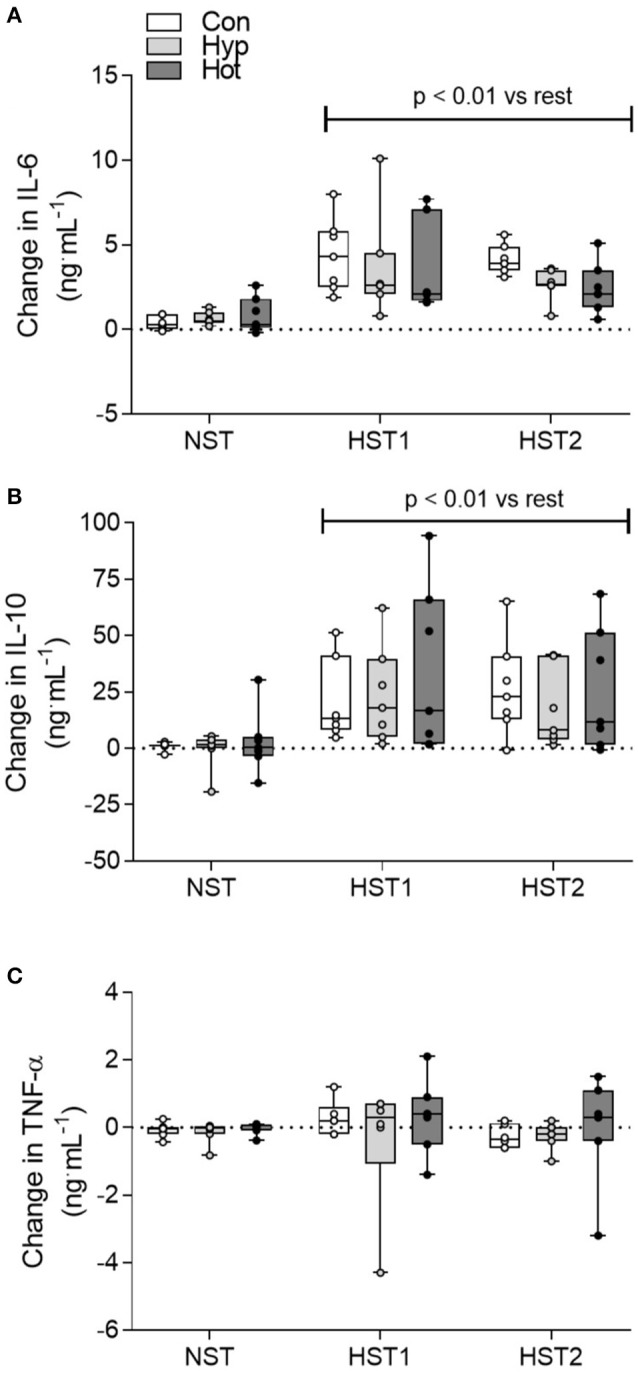
Delta change (ng.mL^−1^) in IL-6 **(A)**, IL-10 **(B)** and TNF-α **(C)** from pre to post-exercise for the NST, HST1, and HST2 trials. Box plots show all individual data points (dots), the 25 and 75th interquartile ranges (boxes), and the median (mid-line). Whiskers illustrate the highest and lowest value. *P*-values indicate differences relative to the pre-trial resting value within each experimental group and between normoxic and hypoxic conditions.

Time × trial interaction effects were observed for IL-6 (*f* = 18.513, *p* < 0.001; Figure 4A) and IL-10 (*f* = 15.037, *p* < 0.001; Figure 4B). Data for TNF-α are shown in Figure 4C. Following the NST plasma IL-6 (pooled data for *n* = 21; +0.62 [0.34–0.91] ng^.^mL^−1^) and IL-10 (+1.1 [−2.78–4.97] ng^.^mL^−1^) exhibited minimal change (*p* > 0.05). After HST1, IL-6 (pooled data for *n* = 21; +3.87 [2.77–4.96] ng^.^mL^−1^) and IL-10 (+26.15 [15.01–37.30] ng^.^mL^−1^) were elevated from rest (*p* < 0.01), with no main effect for acclimation group (*p* > 0.05). A similar trend was observed after acclimation (HST2), with IL-6 (+3.09 [2.53–3.65] ng^.^mL^−1^) and IL-10 (+23.22 [13.95–32.48] ng^.^mL^−1^) increased following exercise (*p* < 0.01), and no main effect for trial or acclimation group observed (all *p* > 0.05).

**Figure 5:**
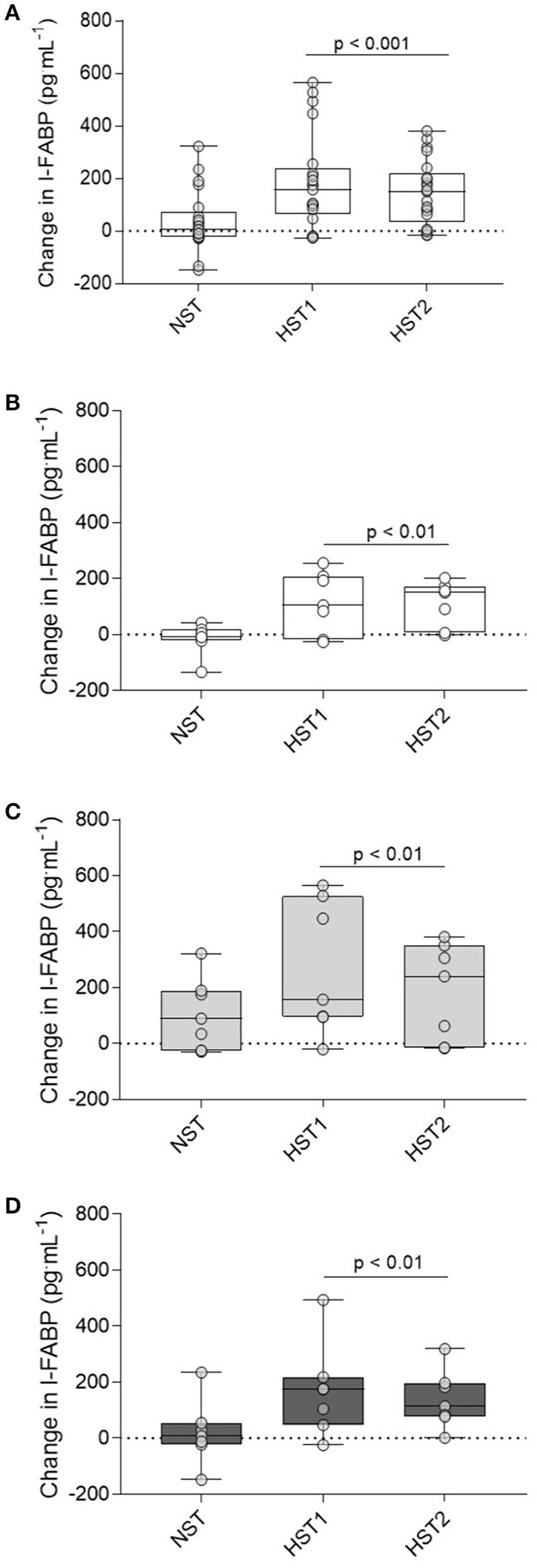
Delta change in I-FABP from pre to post-exercise measurements for the NST, HST1, and HST2 trials for the total participant cohort (*n* = 21; **A**), and the delta change from pre to post-exercise for the CON **(B)**, HYP **(C)**, and HOT **(D)** groups. Box plots show all individual data points (dots), the 25 and 75th interquartile ranges (boxes), and the median (mid-line). Whiskers illustrate the highest and lowest value. *P*-values indicate differences relative to the pre-trial resting values within each experimental group and between normoxic and hypoxic conditions.

Figure 5A shows the fold change from rest for I-FABP after the normoxic trial, pre-acclimation HST, and post-acclimation HST for all 21 participants (A). Figures 5B–D show the fold change from rest for I-FABP for the control (B), hypoxic (C) and heat (D) groups. For absolute I-FABP concentration there was no main effect for trial (*f* = 0.495, *p* = 0.61). There was a main effect for time (*f* = 18.245, *p* < 0.001) and a trial × time interaction (*f* = 10.389, *p* < 0.001). *Post-hoc* analysis showed that pre-exercise I-FABP concentrations were similar at the onset of all trials (*p* = 0.248). No change in post-exercise I-FABP was observed after normoxic exercise (NST pre-exercise; 427 [247–606] pg^.^mL^−1^, post-exercise; 464 [258–670] pg^.^mL^−1^, *p* = 0.153). In contrast, strong evidence for a post-exercise increase in I-FABP was observed following hypoxic exercise (HST1 pre-exercise; 381 [209–553]. post-exercise; 565 [329–800] pg^.^mL^−1^, *p* < 0.001). We found no evidence of blunted I-FABP following either the heat (*p* = 0.44) or hypoxic (*p* = 0.16) acclimation intervention.

## Discussion

The primary aim of the present study was to determine the mHSP72 response throughout both heat and hypoxic acclimation. We hypothesized that both modes of exercise acclimation would induce increases in resting mHSP72. In accordance with this, we show that heat acclimation produces a gradual and progressive increase in resting mHSP72 throughout the 10-day period, whereas the accumulation of resting mHSP72 was only evident on the final day of hypoxic acclimation. These data suggest that the greater physiological strain experienced throughout heat acclimation compared to both control and hypoxic acclimation, is a sufficient stimulus for rapidly enhancing the cytoprotective intracellular HSP72. Hypoxic acclimation at the same absolute work rate (50% normoxic $\overset{\cdot }{V}$O_2_peak), but higher relative work rate (~64 % hypoxic $\overset{\cdot }{V}$O_2_peak) also enhanced resting mHSP72, however this appears to require a longer duration of acclimation. The greater length of time required to increase mHSP72 in the hypoxic group may be due to reduced level of total physiological strain imparted during each session as a result of a smaller thermal stimulus compared to that achieved in the heat group. A secondary aim was to determine whether a period of heat or hypoxic acclimation would reduce intestinal injury and the systemic cytokine response following hypoxic exercise. Moderate evidence for a reduction in post-exercise IL-6 and IL-10 were observed on day 10 of heat acclimation, with an adjustment to a more anti-inflammatory state further indicated by the reduced eHSP72/iHSP72 ratio, though no alterations in intestinal damage markers were observed after the acclimation period. We show that intestinal fatty acid binding protein is increased following moderate intensity hypoxic exercise, and that period of heat or hypoxic acclimation does not attenuate either the post-exercise I-FABP response, or the post-exercise increase in IL-6 and IL-10 after moderate intensity hypoxic exercise.

### Accumulation of monocyte HSP72 during acclimation

The increase in basal intracellular HSP72 reserves is an established outcome of human-heat acclimation (McClung et al., 2008; de Castro Magalhães et al., 2010; Gibson et al., 2015a,b; Lee et al., 2015a, 2016). In the present study we show that monocyte HSP72 begins to increase from the third day of acclimation, and progressively increases over the 10-day acclimation period. These results are similar to those observed in our laboratory, with increased basal mHSP72 found after 3 days of STHA (Lee et al., 2015a). We did not observe a plateau in basal mHSP72 at the end of the 10-day acclimation period, which may suggest that complete cellular adaptation to heat stress, and therefore acclamatory homeostasis was not achieved. During the transition from STHA to LTHA a progressive transcriptional activation and consequent stockpiling of cytoprotective reserves occurs until cellular homeostasis is acquired (Horowitz, 2016). Interestingly, our data may suggest that from a cellular/cross-tolerance perspective, our participants were still in the STHA phase of adaptation. STHA animals have been shown to have a lower tolerance to novel stressors (Assayag et al., 2010, 2012; Yacobi et al., 2014) than their more efficient LTHA counterparts. Therefore, when assessing HACT effects, confirming participants are in the LTHA phase is experimentally important, yet no method exists to confirm this status in humans. Gibson et al. compared the efficacy of both fixed workload and isothermic heat acclimation regimes on HSP72 and HSP90 gene expression, with no differences in gene expression or physiological responses found across the different heat acclimation regimes (Gibson et al., 2015a). However, the maintenance of daily physiological strain afforded by isothermic protocols make this an attractive methodology for attempting to determine whether a plateau in mHSP72 accumulation occurs. Future work is required in order to determine whether a final “plateau” in mHSP72 accumulation represents the complete transition to LTHA, and is therefore the optimal time period to assess cross-tolerance effects.

In contrast, the hypoxic acclimation period induced a slower accumulation in mHSP72, with increased reserves only observed by the final day of acclimation. The slower accumulation throughout acclimation could be due to the lower total physiological strain experienced by participants and reduced thermal component to the exercise stressor. Greater increases in HSP72 are observed when an increase in deep body temperature >38.5°C is induced (Gibson et al., 2015b), therefore, it is likely a combination of the numerous cellular and molecular stressors elicited by exercise were the inductive stimulus for HSP72 in the hypoxic group (Gibson et al., 2017). A hypoxia-mediated induction of monocyte-expressed HSP72 has been suggested to provide protection to the disturbances to redox balance associated with human sub-maximal aerobic exercise (Taylor et al., 2012). Mechanistically, evidence exists that elevations in oxidative stress are a trigger for increases in HSP72 concentration (Kukreja et al., 1994; Ahn and Thiele, 2003) with similar findings shown after both acute (Taylor et al., 2010) and repeated daily resting hypoxic exposures (Taylor et al., 2011, 2012). These authors suggest the repeated disturbance to redox balance from daily hypoxic exposures may act as a stimulus for elevated HSP72 expression (Taylor et al., 2012).

Exercise intensity is an important factor impacting on redox state in monocytes (Wang et al., 2006). Heavy exercise (~80% $\overset{\cdot }{V}$O_2_max) has been shown to increase the production of reactive oxygen species (ROS), and reduced mitochondrial superoxide dismutase and reduced γ-glutamylcysteinyl (GSH) in monocytes. The increases in monocyte ROS production could theoretically act as a cell-type specific stimulus for monocyte HSP72 induction. However, lower workloads (40 and 60% $\overset{\cdot }{V}$O_2_max), which are similar to the relative intensity experienced by participants in the present study (~64% hypoxic $\overset{\cdot }{V}$O_2_peak), were shown not to increase monocyte ROS production (Wang et al., 2006). More recently, daily 2 h exercise bouts at a moderate exercise intensity (50% $\overset{\cdot }{V}$O_2_peak) over a 10 day period enhanced antioxidant capacity, thereby blunting any hypoxia related oxidative stress (Pialoux et al., 2009), a finding that has been corroborated by others using low-to-moderate intensity exercise protocols (Debevec et al., 2016, 2017). In the present investigation relative exercise intensity was not matched between the control group and the hypoxic group, therefore the contribution of a higher relative workload cannot be separated from the hypoxic stimulus. Two experimental approaches could be employed to mitigate this: The incorporation of a matched relative workload control condition would better delineate the contribution of increased relative exercise intensities verses hypoxia *per se*. Alternatively, a passive heating and passive hypoxic acclimation protocol would remove any potential exercise intensity confounds by removing the multifaceted stimuli caused by exercise, thereby allowing the separate effects of each environment to be studied. It should also be acknowledged that an elevated rectal temperature was observed in the hypoxic acclimation group on days 1–6 of acclimation (peak T_rectal_ = 38.1–38.2°C). Therefore, the increase in mHSP72 observed in this group may simply reflect an elevated temperature, albeit to a lesser degree to that observed in the heat group. In addition, the present investigation does not allow for the separate effects of relative workload and thermal stimulus to be compared.

### Heat or hypoxic acclimation does not reduce intestinal damage following hypoxic exercise

In the present study we were unable to demonstrate an acclimation mediated reduction in intestinal barrier damage, as assessed by I-FABP. I-FABP is a robust biomarker of enterocyte damage and intestinal injury, and correlates well with the lactulose/rhamanose sugars gut permeability test (March et al., 2017; Pugh et al., 2017). No change in post-exercise I-FABP was observed on the final day of acclimation relative to day 1, and no change was observed following hypoxic exercise after the acclimation intervention. As such, the primary finding from this aspect of the study is that moderate exercise in hypoxic conditions (reducing arterial oxygen saturation to <85%) is sufficient to induce enterocyte damage and increase circulating I-FABP. However, this data needs confirming with the inclusion of a matched relative exercise intensity control group, as the effects of increasing exercise intensity are known to induce greater intestinal damage (Pires et al., 2016).

A HSP72 mediated inhibition of NF-κB translocation into the nucleus of intestinal epithelial cells has been demonstrated to reduce the synthesis of pro-inflammatory cytokines (Dokladny et al., 2010). This action limits stress-mediated inflammation by preventing endotoxin translocation into the portal circulation, attenuating systemic inflammatory responses, and is another mechanism by which HSP72 accumulation may protect the intestinal barrier (Kuennen et al., 2011; Zuhl et al., 2014). The lack of any observed positive effect on enterocyte damage following the acclimation mediated increase in mHSP72 may be explained by the comparatively short hypoxic exposure period employed (60 min) not being sufficiently stressful to induce severe disturbances to gastrointestinal permeability and pro-inflammatory responses. By employing a more intense hypoxic exercise bout (e.g., >80% $\overset{\cdot }{V}$O_2_max), which is known to severely compromise the epithelial wall and increase gut permeability (Sessions et al., 2016; March et al., 2017), it is possible that any beneficial effects of acclimation induced mHSP72 may be more readily observed. Alternatively, extending the exercise duration to be in line with those observed in occupational or military settings may also warrant additional investigation when determining whether heat/hypoxic acclimation can maintain intestinal permeability. It should however be noted that prolonged resting hypoxic exposures of 3 days (3,300 to 4,392 m; Kalson et al., 2010), and 3 weeks (2,800 to 3,400 m; Mekjavic et al., 2016) have not demonstrated a hypoxia-mediated effect on gastrointestinal permeability or blood flow. Therefore, the relative contribution of both hypoxia and exercise intensity on gut permeability require further investigation.

In the present study, IL-6 and IL-10 were elevated to a similar extent following day 1 of both heat and hypoxic acclimation, with the increase in each being greater than observed in the control condition. The results for IL-6 and IL-10 in the heat group are similar to those observed after a session of isothermic heat acclimation in which T_core_ was clamped at 39.0°C for the final 50 min of a 100-min protocol (Kuennen et al., 2011). The increase in post-exercise IL-6 and IL-10 in the hypoxic group is in accordance with Caris et al. (2016), and could reflect the greater exercise intensity and increased reliance on glucose as a substrate, with IL-6 released from the muscle in order to maintain glucose metabolism (Carey et al., 2006). On the final day of acclimation, there was no alteration in the post-exercise IL-6 or IL-10 in either the control or hypoxic group. However, we provide weak-to-moderate evidence for reductions in both IL-6 and IL-10 following heat acclimation, and a concomitant shift in the ratio of eHSP72 and iHSP72 which further indicates a shift toward an anti-inflammatory status (Krause et al., 2015). However, despite this shift toward an anti-inflammatory state in the heat-acclimation group, no differences in the systemic cytokine response were observed following the post-acclimation hypoxic exercise bout. We acknowledge that the relatively small sample size within each group may have limited the statistical power, and therefore ability to detect small changes in cytokine balance after acclimation, is a limitation of the present study. Further experiments are therefore warranted in order to confirm our observations.

The functional relevance of HSP72 during human heat acclimation was first indicated by using quercetin, a standard laboratory blocker of heat shock transcription factor-1 (HSF-1; Kuennen et al., 2011). By supplementing with quercetin throughout the acclimation phase, Kuennen and colleagues were able to disrupt the normal cellular accumulation of HSP72 in PBMCs. The blunted HSP72 response was associated with impairments to gastrointestinal barrier function, as determined by the lactulose/rhamanose sugars test. The inhibition of HSP72 accumulation and impaired post-acclimation GI function and endotoxin translocation into the systemic circulation disturbed the pro-inflammatory and anti-inflammatory cytokine profiles, which may be indicative of an overactive NF-κB system (Kuennen et al., 2011). In contrast to heat acclimation, the functional relevance of HSP72 in the context of hypoxic acclimation has yet to be fully elucidated. HSP72 has been shown to increase the stability of hypoxia-inducible factor-1a (HIF1a), and may improve both erythropoietic (Taylor et al., 2011) and angiogenic responses (Shiota et al., 2010; Bruns et al., 2012) which are key adaptive responses to hypoxia (Levine and Stray-Gundersen, 1997; Vogt et al., 2001). HSP72 has recently been identified as a specific regulator of both angiogenesis (Bruns et al., 2012; Kim et al., 2016) and erythropoiesis (Ribeil et al., 2004, 2007). However, at this time these proposed mechanisms lack experimental support in human studies, and therefore functional role of increased HSP72 during repeated hypoxic exposures cannot be causally determined. In order to clearly determine role played by increasing HSP72 in the development of heat/hypoxic acclimation and the conferring of cross-tolerance effects, methods that manipulate intracellular HSP72 content are required. Pharmacological interventions that augment HSP72 (e.g., quercetin; glutamine; O-(3-piperidin 2-hydroxy-1-propyl) nicotinic amidoxime-BGP-15) would allow for independent effects of the physiological and cellular adaptations in cross tolerance, and the functional significance of HSP72 in the context of hypoxic acclimation, to be investigated (Gibson et al., 2017).

## Conclusions

This study presents data which show that heat acclimation per-se is a more time efficient means of increasing constitutive stores of monocyte HSP72 when compared to the same period of hypoxic acclimation. In contrast hypoxic acclimation produced a slower accumulation of mHSP72. Although we provide further evidence for heat-hypoxic cross-tolerance, our data show that the benefits of HACT do not affect pro/anti-inflammatory balance and nor do they protect against enterocyte damage. Alternative acclimation modalities, in which the daily stress stimulus is maintained via core temperature clamping may prove to be more effective in preserving intestinal integrity. An intriguing consequence of this experiment is the suggestion that 10 days of fixed work acclimation did not provide full cytoprotective adaptation and that, at least from a cellular perspective, acclimation homeostasis had yet to be achieved. Further studies are therefore warranted to determine the optimal heat “dose” in order to maximize the constitutive HSP72 reserves and potentiate the potential for cross-tolerance effects. From a practical perspective, heat acclimation is a more time-efficient and accessible method for enhancing cytoprotection than hypoxic acclimation. The application of HACT as a means to enhance hypoxic tolerance and operational effectiveness in human occupational, military, and sporting scenarios requires further study, but remains an interesting prospect.

## Ethics statement

This study was carried out in accordance with the recommendations of Coventry University local Ethics Committee, with written informed consent obtained from all subjects. All subjects gave written informed consent in accordance with the Declaration of Helsinki. The protocol was approved by the Coventry University local ethics Committee.

## Author contributions

BL contributed to the conception, data collection sample analysis, manuscript preparation CT contributed to the conception, data collection, manuscript preparation.

### Conflict of interest statement

The authors declare that the research was conducted in the absence of any commercial or financial relationships that could be construed as a potential conflict of interest.
